# Circadian Clock Genes Modulate Immune, Cell Cycle and Apoptosis in the Diagnosis and Prognosis of Pan-Renal Cell Carcinoma

**DOI:** 10.3389/fmolb.2021.747629

**Published:** 2021-12-16

**Authors:** Shuwen Liu, Yongxian Cheng, Shaoxiang Wang, Huiyu Liu

**Affiliations:** ^1^ Institute for Inheritance-Based Innovation of Chinese Medicine, School of Pharmaceutical Sciences, Health Science Center, Shenzhen University, Shenzhen, China; ^2^ College of Physics and Optoelectronic Engineering, Shenzhen University, Shenzhen, China; ^3^ Guangdong Key Laboratory for Functional Substances in Medicinal Edible Resources and Healthcare Products, School of Life Sciences and Food Engineering, Hanshan Normal University, Chaozhou, China

**Keywords:** circadian clock, pan-renal cell carcinoma, immunity, cell cycle, apoptosis, prognosis

## Abstract

**Background:** Pan-renal cell carcinoma (pan-RCC) is mainly divided into renal clear cell carcinoma (KIRC), renal papillary cell carcinoma (KIRP), and chromophobe cell carcinoma (KICH). Pan-RCC is a common malignant neoplasm with a high incidence and poor prognosis. Several studies have demonstrated a close association between cancer development and circadian rhythms; however, the clinical significance and molecular mechanism of the clock gene remain unclear in pan-RCC.

**Methods:** In this study, we systematically characterized the alterations of 15 well-known clock genes of three types of kidney cancer. Bioinformatics methods, including differential expression analysis, survival analysis, signing pathway analysis, co-expression network analysis, and drug sensitivity analysis were used to study the diagnosis, prognostic role, and mechanism of clock genes.

**Results:** Thirteen rhythmic genes fluctuated in circadian rhythm in the kidney tissue of mice, and the opposite trend of these rhythm phases was also found in baboons. There are twelve clock genes that were differentially expressed in at least two types of RCC, of which *NR1D1, DBP, BHLHE40, CRY1*, and *CLOCK* had the same trend in RCC. Changes in clock control genes may be regulated through methylation, copy number, and mutations. Five rhythmic genes, including *PER2, DBP, PER3, CRY2, and RORA,* have significant prognostic role in patient survival in at least two types of kidney cancer. Immune infiltration analysis showed that the expression of these rhythmic genes related to prognosis was positively correlated with the infiltration levels of CD4 and CD8 T cells. Pathway analysis suggests that the clock genes is widely related to cancer-related signaling pathways, such as apoptosis, cell cycle, and other pathways. The PPI network showed that circadian genes are closely linked to cancer-related genes such as *HIF-1A*, *TP53,* and *ERBB2.* Moreover, clock gene expression is correlated with the sensitivity of anticancer drugs such as bleomycin and methotrexate in pan-RCC.

**Conclusion:** Taken together, the abnormal expression of biological clock genes plays an important role in the clinical prognosis of RCC through immunity, cell cycle, and apoptosis. These findings provide a reliable basis for the diagnosis, prognosis, and drug guidance for RCC.

## Introduction

Renal cell carcinoma (RCC) is the deadliest of all urogenital tumors and is the third most common cause of prostate and bladder cancer (Spadaccino et al., 2020). Although collecting duct carcinoma and unclassified carcinoma occur in the kidneys, the major pathological types of pan-RCC are divided into renal clear cell carcinoma (KIRC, 80%), renal papillary cell carcinoma (KIRP, 10–15%), and chromophobe cell carcinoma (KICH, 5%) (Lopez-Beltran et al., 20092009; Muglia and Prando, 2015). Owing to the insidious incidence of kidney cancer, there are no typical clinical symptoms and specific diagnostic signs in the early stage; 17% of patients are already in an advanced stage or metastatic kidney cancer at the time of initial diagnosis (Capitanio and Montorsi, 2016). However, advanced or recurrent renal cancer is not sensitive to chemotherapy and radiotherapy, and the 5-year survival rate is only 20% or less (Posadas et al., 2017). Few sensitive and specific pan-RCC biomarkers for early diagnosis have been clinically validated and can be used routinely. Thus, the discovery of novel biological markers in the pathogenesis of pan-RCC has important clinical significance for the diagnosis and prognosis of the disease.

Circadian clocks are widespread in nature and mainly depend on the biological clock system of the organism and the complex regulatory network of clock control genes. In mammals, clock control genes play an important role in maintaining physiological homeostasis in tumor pathways, such as DNA injury and repair, cell apoptosis, cell proliferation, and migration (Dumbell et al., 2016; Tahara and Shibata, 2016; Pavlova, 2017). When circadian rhythms are interrupted, the risk of several types of cancer is significantly increased, including prostate, colon, endometrial, liver, pancreas, kidney, lung, and breast cancers (El-Athman et al., 2018; Koronowski et al., 2019; Wendeu-Foyet et al., 2019; Wang et al., 2020). Early studies showed that disruption of the circadian system could promote hepatocarcinogenesis through chronic jet lag-driven gene dysregulation and liver metabolic dysfunction (Kettner et al., 2016). Epidemiological studies have also found that women on shift work have a significantly higher breast cancer incidence than women with normal working hours (Kamdar et al., 2013). Disruption of circadian rhythms not only increases the risk of tumor pathogenesis, but also promotes the progression of existing tumors and affects disease diagnosis and prognosis. Interestingly, individual genes of the circadian clock, such as BMAL1, also play an important role in controlling breast cancer (Kwon et al., 2020). Aberrant expression of PER2 promotes the progression of oral squamous cell carcinoma, and upregulated expression of NR1D1 may promote the development of renal clear cell carcinoma (Guo et al., 2020; Zhou et al., 2020). At present, only one study has reported abnormal expression and associated mechanisms of circadian rhythm genes in KIRC; the expression, survival, and molecular mechanism of clock genes in pan-RCC are not clear (Zhou et al., 2020).

In the current study, we observed differential gene analysis of normal and three different types of renal cancer tumor samples in the database to obtain differentially expressed rhythm genes, and further explore the possible relevant mechanisms of differential expression of rhythm genes through methylation and SNV, CNV analysis. Next, the related rhythm genes CRY2, DBP, PER2, RORA, and PER3 affecting the prognosis of pan-RCC were obtained by survival analysis, and analyzed the prognostic mechanism of five rhythm genes regulating pan-RCC from four aspects: immune infiltration, tumor related pathways, co-expression and drug sensitivity. It was found that circadian clock genes can affect the prognosis of pan-RCC by regulating immune, cell cycle and apoptosis pathways. The study of clock genes may help to better understand the prognostic mechanism of pan-RCC, providing novel and valuable biomarkers to benefit patients with kidney cancer.

## Materials & Methods

### Data Sets and Data Availability

Patients with pan-RCC samples collected from The Cancer Genome Atlas database (TCGA: https://portal.gdc.cancer.gov/), including 89 KICH samples (65 cancer tissues and 24 adjacent cancer tissues), 611 KIRC samples (539 cancer tissues and 72 adjacent cancer tissues), and 321 KIRP samples (289 cancer tissues and 32 adjacent cancer tissues). We downloaded the gene expression file, clinical information, illumina human methylation, copy number variation, and single nucleotide variation in pan-RCC patient samples, and eliminated missing values. The expression of circadian genes in normal renal tissues was obtained from Genotype-Tissue Expression (GTEx: https://gtexportal.org).

### Gene Expression, Methylation, CNV, SNV Analysis

We used edgeR package in R (version 4.0.2) to perform the differential expression analysis. The fold change (FC), P value and False Discovery Rates (FDR) (or adjusted P value) of each gene were obtained. Adjusted *p* < 0.05 genes were defined as significantly expressed genes (DEGs). We then drew the heat map with the “pheatmap” package. Student’s t-test was used to analyze methylation differences between tumor and normal samples, and FDR <0.05 was considered significant. The correlation between methylation and gene expression in the pan-RCC was studied using Spearman correlation analysis. Copy number variation (CNV) is divided into heterozygous and homozygous CNV, indicating that the variation occurs on only one chromosome or both. Only rhythm genes with CNV >5% changes were included in the analysis, and the correlation between CNV and mRNA levels was studied using Pearson correlation analysis. We used the R package maftools (https://bioconductor.org/packages/release/bioc/html/maftools.html) to draw a single nucleotide variation (SNV) summary and an oncoplot waterfall plot.

### Survival Analysis

The patients’ clinical data were matched with the RNA-seq data of cancer samples. Samples without clinical information and samples with an overall survival time of less than 30 days in clinical information were excluded from the study. The Kaplan-Meier method was used to analyze gene expression in tumor tissues and the overall survival rate of patients using R software, and survival analysis of clock genes was carried out. The survival curve was drawn to show the difference in survival rates between the high and low expression groups. The P value was calculated using the log-rank test, and *p* < 0.05 was considered a significant difference. We studied the correlation between circadian rhythm genes and clinical characteristics. SPSS (version 23.0; IBM, Armonk, NY, USA) was used for statistical analysis. *p* < 0.05 indicated a correlation.

### Immune Infiltrate Analysis

The TIMER algorithm was used to estimate the association of rhythm genes with tumor immune infiltration (CD4T cells, CD8T cells) in pan-RCC based on the TIMER database (https://cistrome.shinyapps.io/timer/). P*-*values were corrected for tumor purity.

### Pathway Activity and PPI Analysis

The reverse phase protein array (RPPA) data were downloaded from The Cancer Proteome Atlas (http://tcpaportal.org/tcpa/download.html), and protein expression was normalized by standard deviation. We used the sum of the relative protein levels of all positive regulatory components to subtract the sum of the relative protein levels of all negative regulatory components. The pathway activity score (PAS) of 10 cancer-related pathways in each pan-RCC sample was calculated. According to rhythmic gene expression, the tumor samples were divided into high expression group and low expression group, following which, Student’s t-test was used to calculate the difference in pathway scores between the two groups, and *p* < 0.05 was considered statistically different. The expression of genes with statistically significant differences was positively correlated with the pathway activity score, indicating that genes had an activation effect on the pathway; conversely, the negative correlation indicated that genes had an inhibitory effect on the pathway. Based on DisGeNET database (http://www.disgenet.org), according to the score, we extracted the top 30 genes related to renal cell carcinoma. The protein-protein interaction network of rhythm genes and pan-RCC-related genes was drawn using Cytoscape.

### Drug Sensitivity Analysis

Based on the data of drug sensitivity and gene expression profile of tumor cell lines in the Genomics of Drug Sensitivity in Cancer (GDSC: https://www.cancerrxgene.org/), the correlation between rhythm genes and small molecule/drug sensitivity in pan-RCC was studied by Spearman correlation analysis.

## Results

### The Expression Pattern of Circadian Rhythm Genes in the Kidney of Rodents and Primates

**FIGURE 1:**
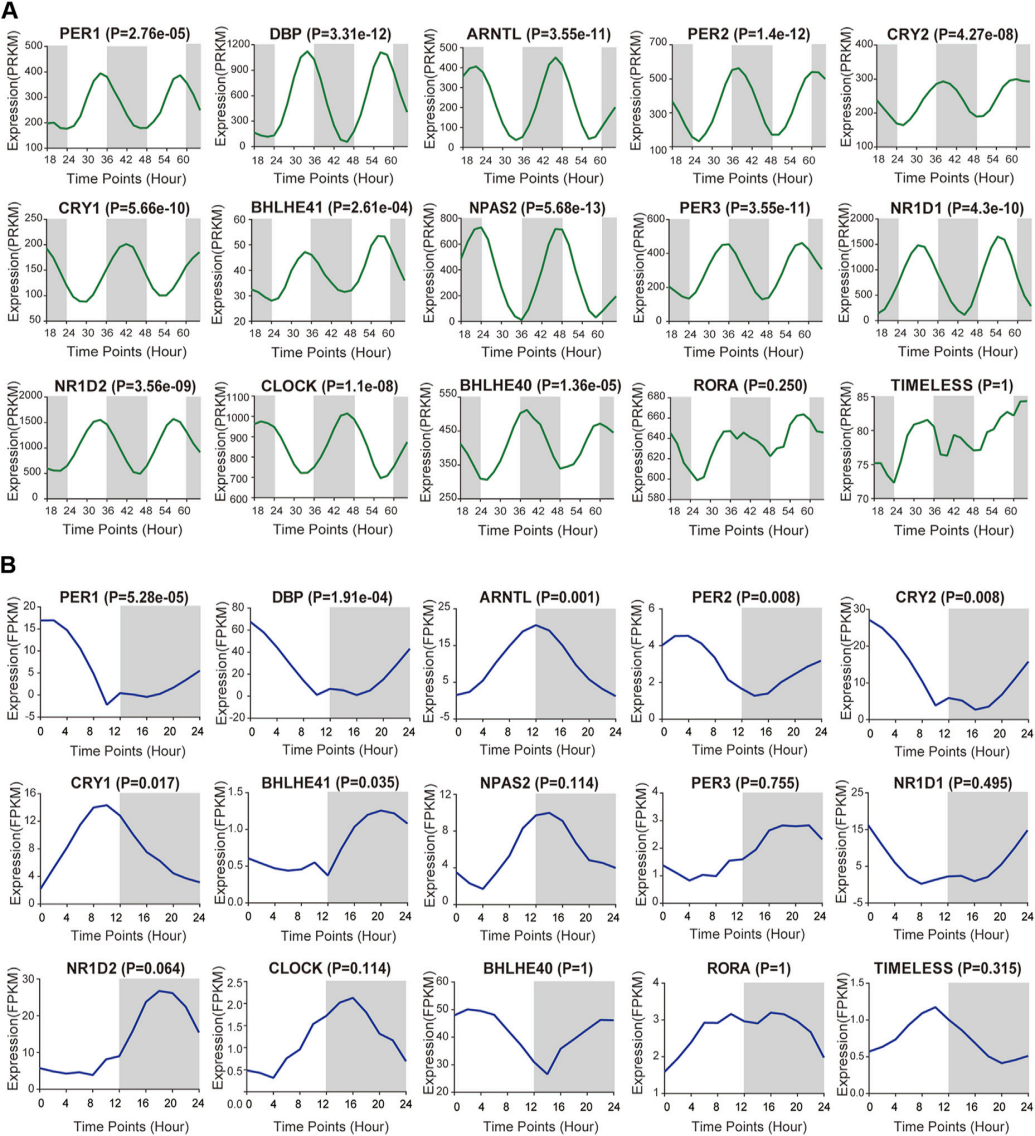
Basic expression of core clock gene in kidney tissues. **(A,B)** RNA-Seq derived from two GEO databases (*Mus* GSE54652, Baboon GSE98965) was used to calculate the expression profiles of core rhythm genes at different time intervals in the renal tissues of mice and baboons, including NPAS2, PER2, DBP, ARNTL, PER3, NR1D1, CRY1, NR1D2, CLOCK, CRY2, BHLHE40, PER1, BHLHE41, RORA and TIMELESS.

To achieve a systematic understanding of the circadian clock in pan-RCC, we first selected 15 well-known circadian genes in this study, namely; NPAS2, PER2, DBP, ARNTL, PER3, NR1D1, CRY1, NR1D2, CLOCK, CRY2, BHLHE40, PER1, BHLHE41, RORA, and TIMELESS (Qiu et al., 2019). RNA-Seq derived from two GEO databases (*Mus* GSE54652, Baboon GSE98965) was used to calculate the expression profiles of circadian rhythm genes at different time intervals in the renal tissues of rodents and primates and to observe the fluctuations of these genes (Zhang et al., 2014; Mure et al., 2018). The results showed that, except for RORA and TIMELESS, the 24-h expression of the remaining 13 clock genes in the mouse kidney was rhythmic (*p* < 0.05, Figure 1A). The 24-h expression of PER1, DBP, ARNTL, PER2, CRY2, CRY1, and BHLHE41 genes in the baboon kidney was rhythmic (*p* < 0.05, Figure 1B). The difference in the peak phase of expression of the same clock gene between mice and baboon kidneys was approximately 12 h. ARNTL and PER1 showed peaks of expression in the baboon in the evening and morning, respectively, whereas in mice, the peak phases occurred in the morning and evening, respectively. Moreover, cancer tissues demonstrate a rhythm similar to that of the corresponding normal tissues (Comas et al., 2014). Therefore, the rhythmic expression of core clock genes in normal kidney tissue presented here could indirectly reflect the expression patterns of core clock genes in pan-RCC.

### The Expression of Circadian Clock Genes is Altered in Pan-RCC

**FIGURE 2:**
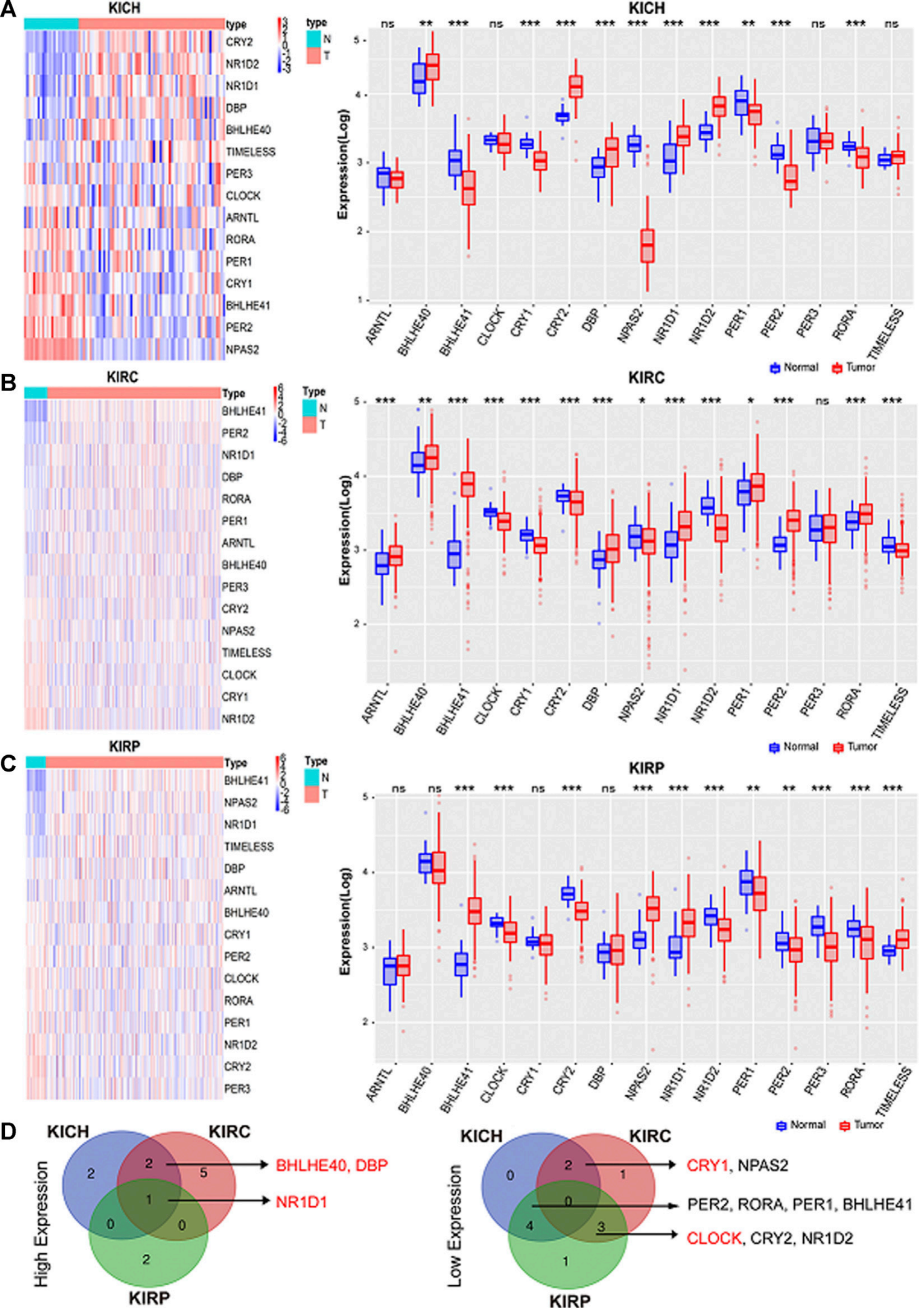
Core circadian clock genes are dysregulated in cancers. **(A–C)** Expression patterns of the clock control genes were shown by heatmaps and boxplots. Scaled expression values in the heatmap are color-coded according to the legend on the right, red represents high expression and blue represents low expression. Here, columns represent patients and rows represent genes, the color bar above the heat map represents the sample groups, and pink indicates cancer tissue, and green represents adjacent normal tissue. The red and blue boxes represent tumor tissues and normal tissues, respectively. *p < 0.05; **p < 0.001; ***p < 0.001. **(D)** The intersection of high expression and low expression clock control genes in pan-RCC. KIRC: Renal clear cell carcinoma. KICH: Chromophobe cell carcinoma. KIRP: Renal papillary cell carcinoma.

We analyzed changes in the mRNA levels of the above-mentioned rhythmic genes in paracancerous normal tissues and pan-RCC. Differential expression analysis showed that the expression of these clock genes in different types of RCC changed. Expression patterns of the clock genes are shown by heatmaps and boxplots (Figures 2A–C). There were 12 clock genes that were differentially expressed in at least two types of RCC, of which NR1D1, DBP, BHLHE40, CRY1, and CLOCK had the same trend in RCC (Figure 2D). Compared with the adjacent normal tissues, the expression of NR1D1, DBP, and BHLHE40 was increased, while the expression of CRY1 and CLOCK was decreased in cancer tissues. Among these different rhythm genes, NR1D1 was upregulated in all three databases, while the expression of BHLHE40 and CLOCK was the highest and lowest in normal kidney tissues, respectively (Supplementary Figure S1A,B). The correlation heat map provided us with an internal correlation of these genes. For instance, DBP and NR1D1, PER1, and PER2 were all significantly positively correlated in the three databases (Supplementary Figures S2A–C). These results may help us understand them more comprehensively.

**FIGURE 3:**
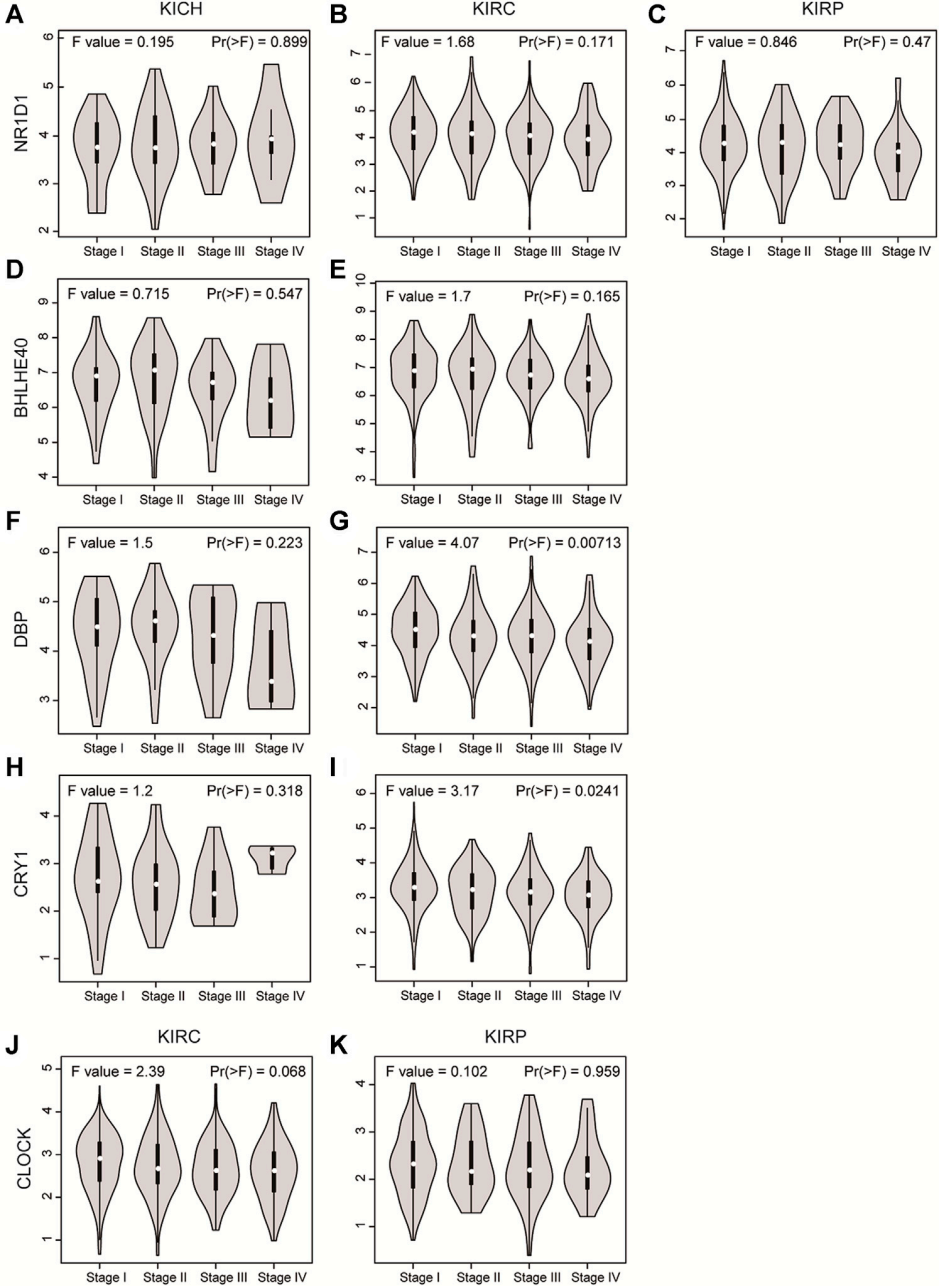
The violin plot showed the expression level of NR1D1, BHLHE40, DBP, CRY1 and CLOCK in different stages of pan-RCC. **(A–C)** NRID1 expression in the stage of pan-RCC. **(D,E)** BHLHE40 expression in the stage of KICH and KIRC. **(F,G)** DBP expression in the stage of KICH and KIRC. **(H,I)** CRY1 expression in the stage of KICH and KIRC. **(J,K)** CLOCK expression in the stage of KICH and KIRC. The parameters were listed in the upper.

**FIGURE 4:**
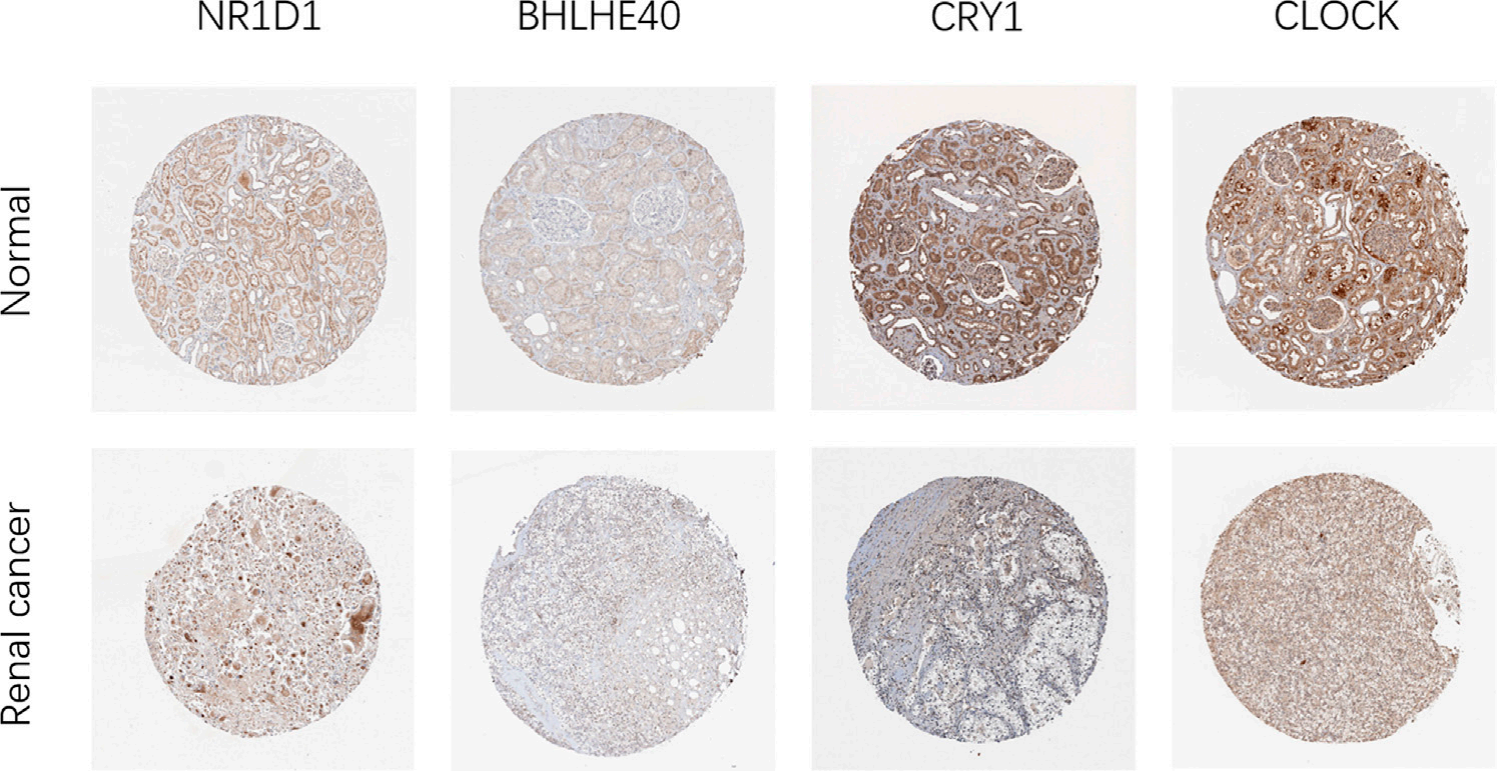
Representative IHC images of NR1D1, BHLHE40, CRY1 and CLOCK expression in normal renal tissues and renal cancer tissues in the Human Protein Atlas.

Next, we analyzed the expression of these genes in tumor samples matched with normal samples, and excluded patient samples treated with some drugs. The results of paired differential analysis were consistent with those of previous differential expression analysis (Supplementary Figures S3A–J). We also analyzed whether there were differences in the expression of NR1D1, BHLHE40, DBP, CRY1 and CLOCK genes in different stages of pan-RCC. The results showed that there were significant differences in the expression of DBP and CRY1 in KIRC at each stage, indicating that DBP and CRY1 had an effect on the stage of KIRC (*p* < 0.05, Figures 3G,I). While there was no significant difference in the expression of NR1D1, BHLHE40 and CLOCK between different stages of renal cell cancer (*p* > 0.05, Figures 3A–F,H,J,K). Then, immunohistochemistry staining validated from the Human Protein Atlas database revealed that NR1D1 and BHLHE40 protein was increased in renal cancer tissues compared with normal renal tissues, the expression of CRY1 and CLOCK protein was significantly decreased in renal cancer tissues compared with normal kidney tissues (*p* < 0.05, Figure 4).

### The Methylation Level, Copy Number Variation, and Mutation of Circadian Clock Genes in Pan-RCC

**FIGURE 5:**
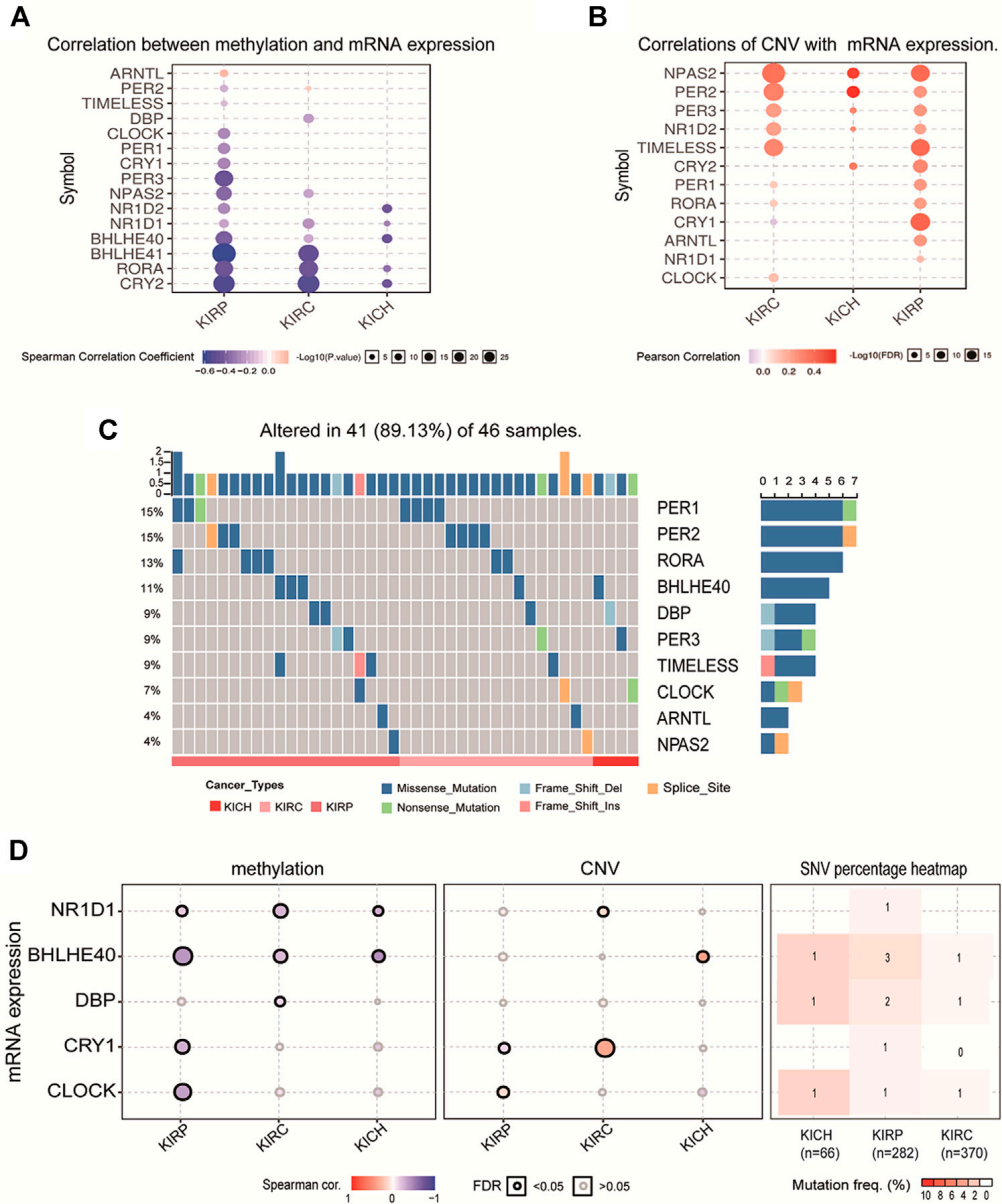
The methylation level, CNV and mutation of clock control genes in pan-RCC. **(A)** Correlation between methylation and mRNA expression in pan-RCC. Here, the blue dots indicate a negative correlation of methylation with mRNA expression, pink dots represent positive correlation, and larger dots confirmed stronger methylation correlation with mRNA expression. **(B)** Correlation of CNV with mRNA expression in pan-RCC. The darker the pink dot indicates a stronger correlation of CNV associated with mRNA expression. FDR<0.05. **(C)** The mutation distribution of rhythm genes in pan-RCC. Here, the columns represent the number of case mutations in patient rhythmic genes, the rows represent rhythmic genes, and the color bars below the figure represent three different types of renal cancer. **(D)** The methylation level, CNV, and mutation of five key differentially expressed genes.

In order to analyze the regulatory factors of circadian clock gene differential expression, we analyzed methylation levels, CNV, and single nucleotide variation (SNV) between normal tissues and pan-RCC. The results showed that in KIRC, the methylation levels of NR1D1, CRY2, and DBP were significantly higher. In contrast, the methylation levels of PER1, BHLHE40, RORA, NPAS2, and BHLHE41 were noticeably lower. In KIRP, the methylation levels of PER3, PER2, and CLOCK were significantly higher. In contrast, the methylation of ARNTL, CRY1, NPAS2, and BHLHE40 was noticeably lower (FDR <0.05, Supplementary Figures S4A). Although genes with differences in methylation vary in different kidney cancer tissues, most of their methylation levels were negatively correlated with mRNA expression (*p* < 0.05, Figure 5A). The results of CNV analysis showed significant heterozygous amplification of TIMELESS, CRY1, and BHLHE41, while PER3, NR1D2, and BHLHE40, are usually heterozygous deletions (Supplementary Figures S4B). In contrast, homozygous deletions and amplifications were extremely low (Supplementary Figures S4B,C). The copy numbers of NPAS2, PER2, PER3, NR1D2, TIMELESS, CRY2, PER1, and RORA were positively correlated with their gene expression, with statistical significance (FDR <0.05, Figure 5B). Mutation analysis of clock gene variation showed that PER1, PER2, RORA, and BHLHE40 are the four genes with a high mutation frequency ( >10%). Of the 46 analyzed patients, 89.13% had at least one mutation (FDR <0.05, Figure 5C; Supplementary Figures S5A). We found that among the five key differentially expressed genes, NR1D1 is mainly regulated by methylation, BHLHE40 is affected by methylation and mutation, DBP may be more susceptible to mutation, CRY1 is mainly regulated by copy number, and CLOCK may be comprehensively regulated by methylation, copy number, and mutation (*p* < 0.05, Figure 5D).

### The Clinical Relevance of Circadian Rhythm Genes in Pan-RCC

**FIGURE 6:**
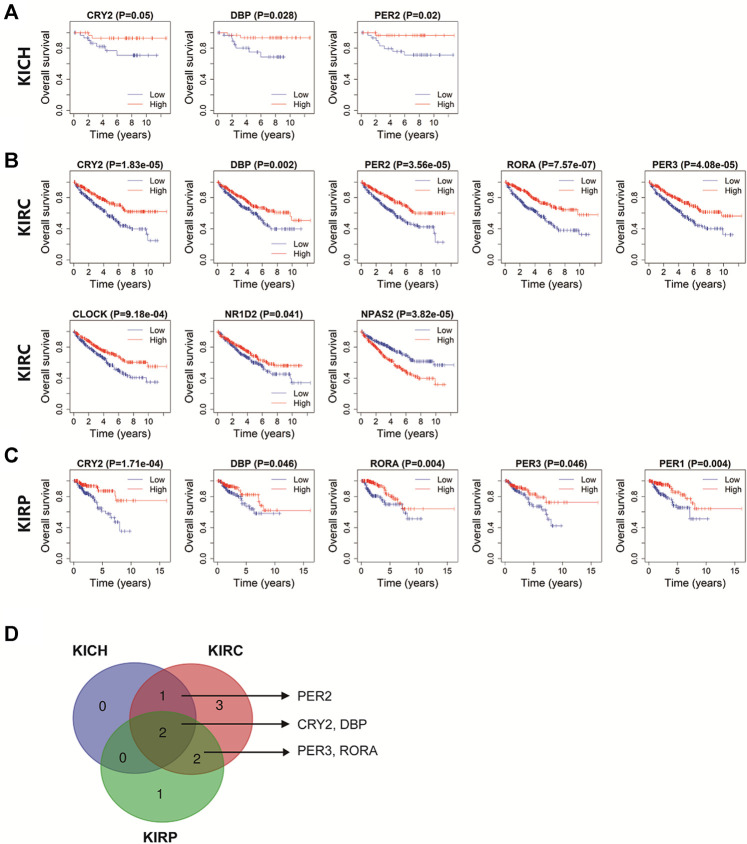
The prognostic value of rhythm genes in pan-RCC. **(A–C)** The prognostic relevance of core clock genes in pan-RCC. Blue lines represent genes low expression in cancer, red lines represent genes high expression in cancer. The abscissa of the survival curve is for the observation time, and the ordinate is for the survival rate. Each point on the curve represents the patient survival at that time point. **(D)** Rhythm genes with significant prognostic value in at least two database, including PER2, CRY2, DBP, PER3, RORA.

The Kaplan-Meier method was used to investigate the prognostic role of core clock gens in pan-RCC. In KICH patients, high expression of CRY2, DBP, and PER2 was significantly associated with a higher likelihood of survival (*p* < 0.05, Figure 6A). In KIRC patients, high levels of CRY2, DBP, PER2, RORA, PER3, CLOCK, and NR1D1 were associated with a higher likelihood of survival, while high expression of NPAS2 was associated with a lower likelihood of survival (*p* < 0.05, Figure 6B). High expression of CRY2, DBP, RORA, PER3, and PER1 were significantly associated with increased likelihood of survival in KIRP patients (*p* < 0.05, Figure 6C). Five core clock genes, CRY2, DBP, PER2, RORA, and PER3, have significant prognostic value in at least two databases, while CRY2 and DBP have prognostic effects in all three databases (Figure 6D), suggesting that they are potential prognostic biomarkers for pan-RCC.

Chi-square test analyses were performed to investigate the relationship between the five prognostic core clock genes and clinical features, including age, sex, TNM stage, grade stage, smoking, bias, and body mass index. The expression of CRY2, PER2, PER3 and RORA was correlated with TNM stage, T stage, and tumor differentiation in KIRC patients (*p* < 0.05, Table.1). CRY2 expression was associated with age and sex, and PER2 expression was associated with TNM staging in KICH patients (*p* < 0.05, Supplementary Table S1). CRY2 expression was related to age, DBP expression was related to age, tumor type, smoking history, and RORA expression was related to TNM stage, obesity index, and tumor type in KIRP patients (*p* < 0.05, Supplementary Table S2).

**TABLE 1:** The relationship between the five prognostic core clock genes and clinical features in KIRC patients.

Variables	CRY2	DBP	PER2	PER3	RORA
	χ^2^(p-Values)	χ^2^(p-Values)	χ^2^(p-Values)	χ^2^(p-Values)	χ^2^(p-Values)
Ages(years)	13.253(0.000^∗∗∗^)	5.409(0.020^∗^)	13.676(0.000^∗∗∗^)	18.866(0.000^∗∗∗^)	21.083(0.000^∗∗∗^)
≤50(114)					
>50(416)					
Gender					
Female(186)					
Male(344)					
TNM stge	18.523(0.000^∗∗∗^)	8.900(0.003^∗∗^)			
I+II(322)					
III+IV (205)					
T stage	15.884(0.000^**∗∗∗**^)	9.484(0.002^∗∗^)	9.484(0.002^∗∗^)	11.847(0.001^∗∗^)	15.884(0.000^∗∗∗^)
T1+T2(340)					
T3+T4(190)					
M stage	8.509(0.004^∗∗^)	6.563(0.010^∗^)	10.224(0.001^∗∗^)	13.295(0.000^∗∗∗^)	18.340(0.000^∗∗∗^)
M0(440)					
M1(80)					
Laterality	4.180(0.041^∗^)				
Right(280)					
Left(249)					
Grade	15.504(0.000^∗∗∗^)		21.779(0.000^∗∗∗^)	17.027 (0.000^∗∗∗^)	11.817(0.001^∗∗^)
G1+G2(241)					
G3+G4(281)					

a *p*< 0.05; **p < 0.001; ***p < 0.001.

### Immune Infiltration Analysis of Circadian Rhythm Genes in Pan-RCC

**FIGURE 7:**
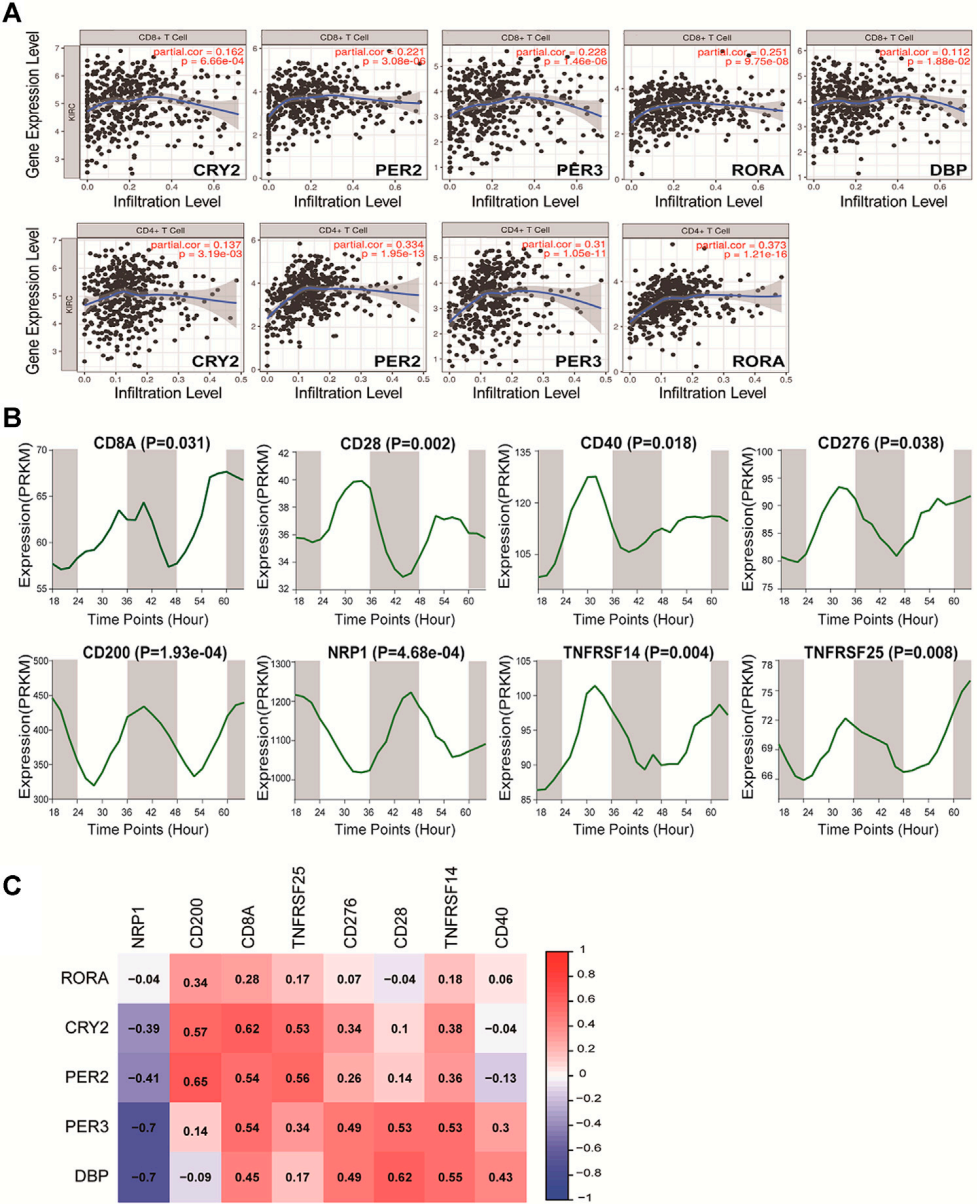
Correlation of five prognostic core clock genes with immune infiltration. **(A)** The five prognostic core clock genes were correlated with CD8 T cells and CD4 T cells. The abscissa represent the infiltration level and the ordinate represent the gene expression level. **(B)** The rhythmic expression patterns of eight immune cell markers at different time intervals in mice renal tissue (*Mus* GSE54652). **(C)** The expression of five prognosis clock control genes was associated with the expression of these key immune genes. Here, columns represent key immune genes and rows represent clock genes, red represents positive correlation between rhythm genes and immune genes, and blue represents negative correlation between rhythm genes and immune genes.

Immune regulation is recognized as one of the most important factors affecting the prognosis of cancer. The correlations between these core clock genes and immune cells in pan-RCCs were explored using the online tool TIMER. Among the five prognostic core clock genes, CRY2, PER2, PER3, DBP, and RORA were positively associated with infiltration levels in CD8+T cells, and CRY2, PER2, PER3 and RORA were positively associated with infiltration levels in CD4+T cells. Conversely, in KIRP, CRY2 and RORA were negatively correlated with the infiltration level of CD8+T cells (Figure 7A; Supplementary Figures S5B), suggesting that the prognostic ability of core clock genes is closely related to the regulation of immune cells. Next, we identified the circadian rhythms of eight immune cell markers, including CD28, CD40, CD276, CD200, NRP1, TNFRSF14, and TNFRSF25, and found that their expression exhibited significant rhythmic expression patterns in mouse renal tissues (Figure 7B). In addition, correlation analysis showed that the expression of five prognostic core clock genes was highly correlated with the expression of these key immune genes (Figure 7C). These results suggest a possible link between the circadian clock and immune infiltration in pan-RCC, and chrono-immunotherapy may serve as a candidate option for future cancer management.

### Pathway Activity and PPI Analysis of Circadian Rhythm Genes in Pan-RCC

**FIGURE 8:**
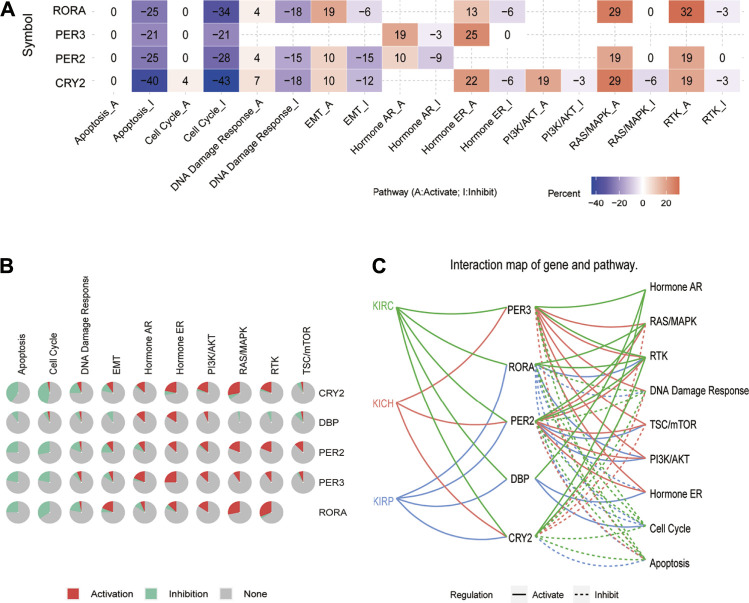
Pathway activity analysis of five prognostic core clock genes in pan-RCC. **(A, B)** The main role of five prognosis core clock genes in tumor-related marker pathways. Here, columns represent tumor-related marker pathways and rows represent genes, red represents rhythm genes activating immune-related pathways, and blue represents rhythm genes suppressing immune-related pathways. **(C)** Links between five prognosis core clock genes and hallmark signaling pathways. Solid lines represent rhythmic gene-activated immune-related pathways, and dashed lines represent immune-related pathways of rhythmic genes.

GSCA was further used to analyze the possible molecular mechanisms involved in core clock genes. The results showed that CRY2, PER2, PER3, and RORA may highly inhibit the cell cycle and apoptosis, and slightly inhibit DNA damage and EMT. In addition, they could possibly activate hormone ER, RAS/MAPK, and RTK pathways to different extents (Figures 8A,B). Specifically, we also found that CRY2 and PER2 inhibited the cell cycle and apoptosis pathways in KICH, KIRC, and KIRP (Figure 8C). These results suggest that disturbance of the biological clock may lead to cell cycle, apoptosis, and other cancer-related pathways, thus affecting the prognosis of patients.

**FIGURE 9:**
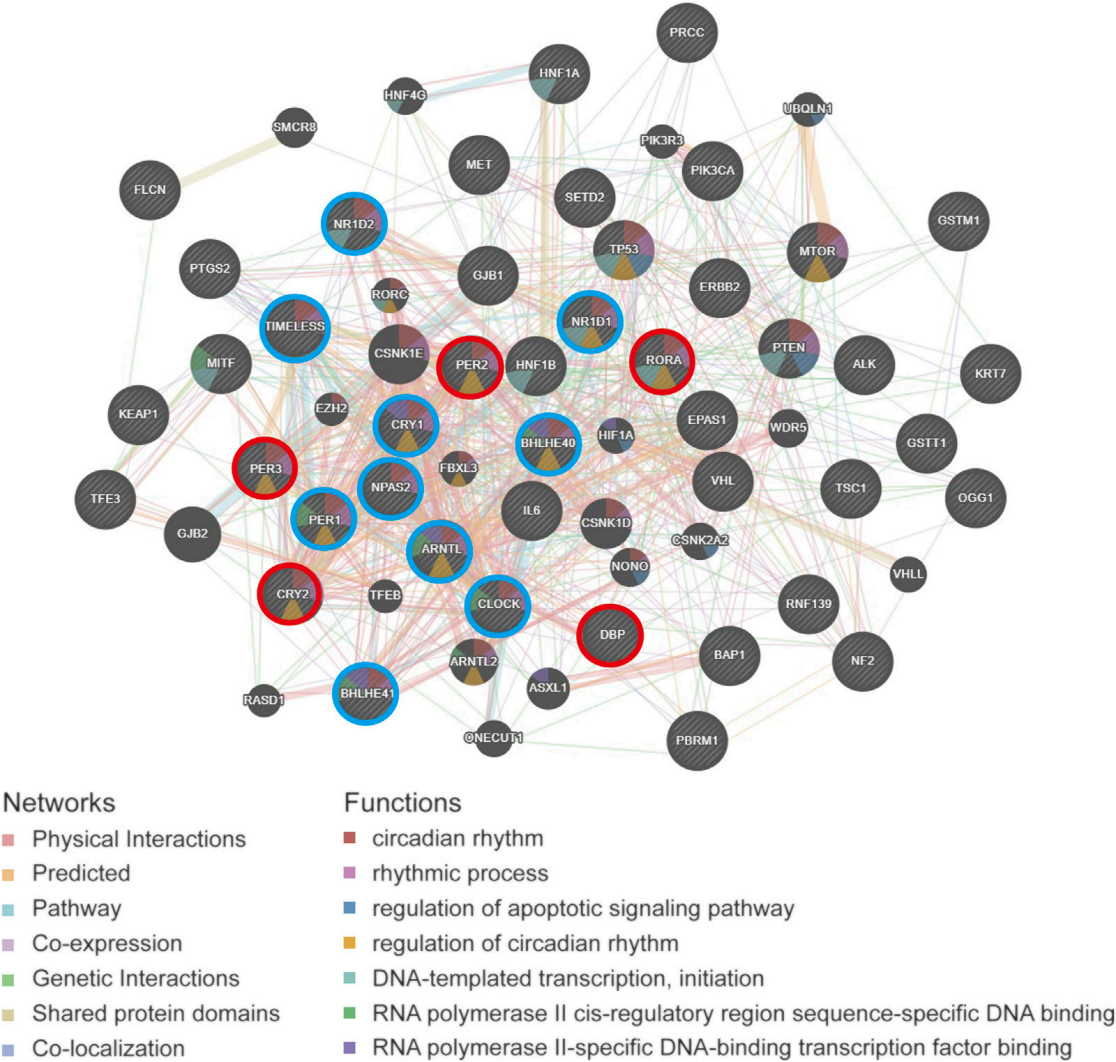
The PPI network analysis of circadian rhythm gene and cancer-related gene of pan-RCC. The PPI network diagram shows the top 30 nodes in terms of correlation, of which five prognostic core clock genes and other clock control genes are circled in red and blue, respectively.

The PPI network diagram shows the top 30 nodes in terms of correlation, of which five prognostic core clock genes and other core clock genes are circled in red and blue, respectively. We found that the circadian genes were associated with oncogenes such as HIF1A, TP53, ERBB2, and IL-6 (Figure 9). These results suggest that clock genes are associated with changes in multiple oncogenic pathways and genes, and that circadian clock disorders can affect tumorigenesis and patient prognosis.

### Drug Sensitivity Analysis of Circadian Rhythm Genes

**FIGURE 10:**
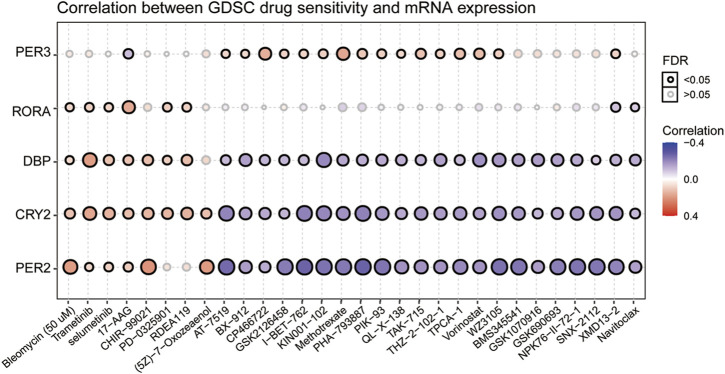
Correlation between GDSC drug sensitivity and six prognostic core clock genes. The correlation between rhythm genes and drug sensitivity in pan-RCC was studied by Spearman correlation analysis. Here, columns represent GDSC drug sensitivity and rows represent clock genes, red represents positive correlation between GDSC drugs sensitivity and rhythm genes mRNA expression, and blue represents negative correlation between GDSC drugs sensitivity and rhythm genes mRNA expression.

To clarify the link between clock genes and existing drug targets in pan-RCC, we analyzed the correlation between biological clock gene expression and drug sensitivity (Figure 10). The results showed that the high expression of RORA, DBP, CRY2, and PER2 might be resistant to bleomycin, trametinib, selumetinib, and 17-AAG. Low expression of DBP, CRY2, and PER2 is considered to be resistant to methotrexate, vorinostat, and navitoclax. The low expression of PER3 may be resistant to 17-AAG, and high expression of PER3 may be resistant to methotrexate and vorinostat. Therefore, according to the biological rhythm of tumor cells, development of individualized time administration schemes for tumor patients may become the development trend of tumor treatment.

## Discussion

Given the circadian regulation of cancer-related physiological systems (e.g., immune response, cell cycle, and apoptosis), chron-therapy may become a promising trend in tumor therapy (Dallmann et al., 2016). The principle of definite timing of administration through therapeutic intervention may represent an innovative strategy to improve the outcome of treatment and the survival rate of potential patients. For advanced or metastatic pan-RCC, the emergence of time immune therapy other than radiotherapy and chemotherapy has been considered an important aspect of anticancer therapy (Ohdo et al., 2019). To evaluate the role of clock control genes in the progression, to find reliable biological targets, and to optimize the diagnosis, prognosis, and treatment of pan-RCC over time, we analyzed the altered expression, mutation, and methylation status of rhythmic genes in patients with RCC. We did this by using a series of statistical and bioinformatics methods to elucidate the relationship between clock control genes expression and patient survival, tumor stage, and subtype. Specifically, we identify the relationship between rhythmic gene expression affecting prognosis and oncogenic pathways, levels of immune invasion, and sensitivity to anticancer drugs. These results suggest that clock control genes play an important role in the diagnosis and prognosis of pan-RCC.

The kidney is the most active tissue with rhythm genes other than liver tissue; therefore, it is necessary to study the relationship between kidney tissue rhythm and cancer (Zhang et al., 2014). In this study, we selected 15 core clock genes that have been extensively studied because of their important role in a variety of cancers (Yang et al., 2017). We found that 13 core clock genes fluctuated in circadian rhythm in the kidney tissue of mice. By analyzing the specific expression patterns of these clock genes in the renal tissues of normal baboons and mice, we agree with other findings that the clock genes described above have rhythmic fluctuations within 24 h. We also agree that the expression of the same clock genes in mice and baboons is opposite; the expression peak phase of each clock component moved ∼ 12 h (Zhang et al., 2014; Mure et al., 2018). Mice an baboon databases differ in that mice detected changes in 48 h renal rhythms, baboons tested changes in 24 h renal rhythms, and we speculate that changes in the 24 h gene were detected due to the higher cost of the baboons from the above fluctuations in the clock gene, we can confirm whether in mice or baboons, most of the clock genes in the kidney were rhythmic.

Circadian rhythm alterations constitute a risk factor for the development of different cancer types, and the occurrence of cancer can also lead to an imbalance in the biological clock system (Okabe et al., 2014; Liu et al., 2019). In this study, differential expression analysis showed that the expression of core clock genes in different types of RCC changed, and CRY1, NR1D1, CLOCK, DBP, and BHLHE40 were differentially expressed in at least two types of RCC. As core clock genes promoter methylation can change the expression state of target genes, and CNV and SNV can change gene expression levels, exploring the changes in core clock genes structure, gene expression, and tumor pathogenesis by observing these indicators has certain clinical significance for the diagnosis and prognosis of kidney cancer (Diskin et al., 2009; Ricketts et al., 2012; Deng et al., 2017). In the present study, we calculated the correlation between core clock genes and methylation levels in normal and pan-RCC tissues. The results showed that the expression of core clock genes was negatively correlated with the methylation of cancer tissue, consistent with the other scholar findings that methylation down-regulated almost all biological clock gene expression in thoracic cancers (Yang et al., 2019). In addition, we concluded that NR1D1 and BHLHE40 may be mainly regulated by methylation, DBP may be more prone to mutations, and CRY1 may be regulated by CNV, confirming that core clock genes may be altered by different mechanisms. Given this fact, it is reasonable to assume that the CNV and SNV of circadian genes may affect cancer susceptibility, cancer cell proliferation, invasion, and response to treatment, as well as the survival of patients. The impact of gene mutations on overall survival helps to assess the relationship between genomic mutations and clinical outcomes(Liu et al., 2019).

Research showed that the dysregulation of a high fraction of clock molecules is associated with the prognosis of patients with KIRC (Zhou et al., 2020). In this study, overall survival (OS) analysis showed that core clock genes including DBP, CRY2, PER2, PER3, and RORA were closely related to the prognosis of pan-RCC patients in at least two databases, while DBP and CRY2 have prognostic effects in all three databases, suggesting that they are potential prognostic biomarkers for pan-RCC. The expression of ARNTL2 was significantly associated with survival time in patients with lung adenocarcinoma, and high ARNTL2 expression predicted poor survival in these patients (Brady et al., 2016). Furthermore, our study confirmed that the expression of core clock genes was closely related to age, sex, tumor type, obesity index, and TNM stage. For instance, CLOCK expression levels increased significantly in human colorectal cancer (CRC) tissues, which are closely related to late TNM staging and positive lymph node metastasis (Wang et al., 2017). Another study confirmed that abnormal expression or deletion of PER1, PER2, and PER3 was associated with poor NSCLC differentiation, lymph node metastasis, and TNM staging (Liu et al., 2014). In brief, different clock gene expression levels may lead to the occurrence of different types of tumors and are also closely related to the prognosis of these cancers.

The composition of immunocytes in the tumor microenvironment is also known to affect cancer prognosis (Ock et al., 2016; Zhou et al., 2020). We investigated the correlation between core clock genes, which are closely related to prognosis and immune cell infiltration. Specifically, we found that CRY2, PER2, PER3, DBP, and RORA were positively associated with infiltration levels in CD8+T cells; CRY2, PER2, PER3 and RORA were positively associated with infiltration levels in CD4+T cells in pan-RCC. In relevant studies on KIRC, they also suggested a strong correlation between the circadian clock and immune cells, including B cells, CD4+T cells, CD8+T cells, neutrophils, macrophages, and dendritic cells (Zhou et al., 2020). Other studies have found that the core negative regulators of core clock genes (PER1, PER2, PER3, CRY1, and CRY2) are downregulated in many tumors, and the decrease in the expression of these core circadian negative regulatory molecules is significantly associated with T cell failure and upregulation of immunosuppressive molecules (Wu et al., 2019). Surprisingly, we found another interesting and important phenomenon in which the expression of immune checkpoint genes CD28, CD40, CD276, CD200, NRP1, TNFRSF14, and TNFRSF25 in normal renal tissue was time-dependent in the database. These immune checkpoint genes were related to five prognosis-related circadian clock genes in RCC. These results suggest a possible link between the circadian clock and immune infiltration, and chrono-immunotherapy may serve as a candidate option for future cancer management.

Here, we found that five rhythm genes closely related to prognosis were associated with the pathways of cell cycle and apoptosis in pan-RCC. The core clock genes extensively affects cancer-related signaling pathways (Sulli et al., 2019). Many studies have shown that circadian clock genes are involved in the regulation of pathways such as cell proliferation, apoptosis, and cell cycles (Kelleher et al., 2014), and there are molecular links with oncogenes and tumor suppressor factors such as Ras and p53 (Gotoh et al., 2016; Lamia, 2017). In accordance with a previous report, another independent group highlighted that the cell cycle pathway mediated the deregulation of the circadian clock in KIRC (Zhou et al., 2020). For example, overexpression of PER2 induces decreased proliferation and increased apoptosis by promoting autophagy in OSCC (oral squamous cell carcinoma) cells (Liu et al., 2020). Cancer cell lines exposed to ionizing radiation (IR) whilst overexpressing PER1 showed increased cell apoptotic sensitivity. In contrast, suppression of PER1 expression by siRNA in IR-exposed cancer cell lines resulted in a reduction in the apoptotic rate (Gery et al., 2006). Moreover, PPI network analysis revealed that circadian genes were associated with ERBB2, TP53, and HIF1A in pan-RCC. For instance, HIF1A increases PER2 transcriptional activity by directly binding to the HRE-like element within the PER2 promoter in RCC lines (Okabe et al., 2014).

Consistent with this finding, we show that clock genes are highly correlated with the sensitivity of most anticancer drugs through multiple pathways. We found that the high expression of RORA, DBP, CRY2, and PER2 might be resistant to bleomycin, trametinib, selumetinib, and 17-AAG. Low expression of DBP, CRY2, and PER2 is considered to be resistant to methotrexate, vorinostat, and navitoclax. Methotrexate mainly suppresses DNA biosynthesis, and we speculate that core clock genes expression is associated with the sensitivity of pan-RCC to the inhibition of methotrexate DNA synthesis. Other studies have confirmed that high BMAL1 expression is associated with increased sensitivity of colorectal cancer cells to oxaliplatin (DNA/RNA synthesis inhibitor) and predicts favorable outcomes for patients treated with oxaliplatin-based chemotherapy (Zeng et al., 2014). Taken together, our results provide strong evidence that the optimal time to deliver drugs to personalize or optimize treatment is important for the treatment of pan-RCC.

Further, the core clock genes expression changes and related mechanisms of KIRC, KIRP, KICH in pan-RCC have also been rarely reported. Liu et al. found that reduced rhythmic gene expression was strongly associated with hypermethylation levels in pan-RCC, with the rhythmic gene index generally significantly reduced in almost all tumors. However, the expression levels of PER1, PER2 in KIRC and CRY2 in KICH were significantly increased in tumor tissues, and they suggested subsequent in-depth investigation of the mechanisms underlying circadian abnormalities in these three renal carcinomas (Liu et al., 2019). Wu et al. performed differential expression analysis of 14 rhythmic genes in 11 cancers, indicating that most negative regulators such as PER1, PER2, PER3, CRY1, CRY2 and NR1D2, were downregulated in pan-cancers, but they mainly targeted these 11 cancers and did not dig into the common expression changes and related mechanisms of rhythm genes in pan-RCC (Wu et al., 2019).

In summary, this study systematically analyzed gene expression levels, methylation, copy number variation, site mutation, OS, infiltrating immune level, immune biomarkers, cancer-related pathways, and drug sensitivity related to clock control genes in pan-RCC. We found that fivecore clock genes, including*, PER2, DBP, PER3, CRY2, and RORA* have significant prognostic ability with regard to patient survival, and are closely associated with immune cell regulation, cell cycle, and apoptosis. Our work strengthens the importance of clock control genes as prognostic biomarkers and their clinical implications for RCC chronotherapy.

## Data Availability

All datasets presented in this study are included in the article/Supplementary Material.
